# Teledentistry Awareness and Perception Among Dentists Managing Pediatric Patients in Kuwait

**DOI:** 10.1089/tmr.2025.0007

**Published:** 2025-04-04

**Authors:** Shurooq Alquhaisan, Mohammad A. Alhasan, Hessa Albader, Saad Alqahtani, Amrita Geevarghese

**Affiliations:** ^1^ Kuwait Board in Pediatric Dentistry, Kuwait Institute for Medical Specializations, Sulaibikhat, Kuwait.; ^2^ Kuwait Board in Pediatric Dentistry, Ministry of Health, Kuwait Institute for Medical Specializations, Sulaibikhat, Kuwait.; ^3^ Dental Public Health Specialist, Ministry of Health, Sulaibikhat, Kuwait.; ^4^ Dental Public Health Specialist, Faculty Development Department, Kuwait Institute for Medical Specializations, Sulaibikhat, Kuwait.

**Keywords:** teledentistry, perception, awareness, Kuwait

## Abstract

**Background:**

Teledentistry integrates digital telecommunication technology with dentistry to enhance the quality, accessibility, and cost-effectiveness of dental care, particularly in remote areas. It improves operational efficiency and access to care, especially for pediatric dental patients.

**Objective:**

This study aims to assess the awareness and perceptions of teledentistry among dentists managing pediatric patients in the government sector in Kuwait.

**Methods:**

A descriptive, cross-sectional study was conducted using a self-administered questionnaire to evaluate awareness and perceptions across three domains: the usefulness of teledentistry for patients and dental practice, its potential to improve practice, and concerns regarding its implementation.

**Results:**

A total of 106 responses were received, yielding a 26.7% response rate. Approximately 48% of respondents expressed concerns regarding the reliability of teledental equipment, patient confidentiality, and obtaining informed consent, as well as the security of sending data online. Despite these concerns, 75% of respondents acknowledged the importance of teledentistry in enhancing dental practice. More than half 56% were unaware of the legal implications of teledentistry in Kuwait. Additionally, over 50% recognized the benefits of teledentistry in clinical training, cost reduction, and time-saving. Respondents also supported the integration of teledentistry into dental education and appointment scheduling.

**Conclusion:**

The study found that pediatric dentists in Kuwait exhibit a high level of awareness and positive perception of teledentistry’s potential to improve dental practice, despite concerns about its technological and legal implications.

## Introduction

Teledentistry, a specialized domain of telemedicine, integrates telecommunications with dentistry, facilitating the exchange of clinical information and images across remote locations for consultations, diagnoses, education, and treatment planning. As an innovative tool, teledentistry offers significant benefits across various dental disciplines, including pediatric dentistry. Literature demonstrates its application across dental specialties without notable advantages for any specific specialty.^1,2^ Teledentistry has the potential to enhance access to oral health care, improve care quality, and optimize the use of health care professionals’ skills. The American Dental Association recognizes two primary modalities of teledentistry: synchronous (real-time) and asynchronous (store-and-forward).^3^ The synchronous modality involves live, two-way interactions between patients, caregivers, or providers using audiovisual telecommunications technology. In contrast, asynchronous teledentistry entails the secure transmission of health information—such as radiographs, photographs, videos, digital impressions, and photomicrographs—to practitioners for evaluation and diagnosis. High-speed internet connectivity is essential to facilitate the data-intensive requirements of this modern system. The primary goals of teledentistry include enhancing dental care quality, reducing costs, improving efficiency, expanding access, and lowering the burden of oral disease.^4,5^

Evidence suggests that dentists generally hold positive attitudes toward teledentistry.^6,7^ For instance, a 2002 study by Stephens and Cook highlighted its efficacy in improving accessibility for consultations.^8^ Similarly, in preventive and pediatric dentistry, teledentistry has proven efficient for screening schoolchildren for early childhood caries, with accuracy comparable to traditional visual/tactile examinations.^9–12^ AlShaya et al. confirmed its reliability in diagnosing incipient caries.^13^ Internationally, attitudes and awareness of teledentistry vary. In India, Boringi et al. reported inadequate knowledge among dental professionals in academic settings.^14^ Conversely, Pradhan et al. found postgraduate dental students had satisfactory awareness and attitudes.^15^ In Australia, Kruger et al. documented broad support among practitioners for integrating teledentistry into current practice.^16^ Similarly, Al-Khalifa et al. noted readiness among dental professionals in Saudi Arabia to adopt teledentistry.^17^ In Kuwait, teledentistry practices are regulated under Chapter 3, Article 24 of Decree 70/2020, which permits remote medical and home health services, leveraging artificial intelligence, advanced technologies, and digital tools under the regulations of the Ministry of Health (MOH). Despite its growing importance, teledentistry faces challenges and barriers. Both governmental and private sectors in Kuwait have begun integrating teledentistry, yet there is limited research examining the awareness and attitudes of pediatric dentists toward its application in health care delivery. Understanding dentists’ perspectives and knowledge is vital for shaping policies, developing effective training programs, and promoting the integration of teledentistry into dental practice to enhance pediatric oral health care delivery.

### Aim

To evaluate the awareness and perceptions of teledentistry among dentists managing pediatric patients in Kuwait’s government sector.

### Objectives

1. To assess dentists’ perceptions of the efficacy of teledentistry in enhancing dental practice and patient outcomes.
2. To evaluate dentists’ awareness of the legal implications of teledentistry in Kuwait.
3. To examine the influence of demographic variables on dentists’ perceptions of teledentistry.

## Methodology

This descriptive cross-sectional study utilized an electronic self-administered questionnaire to evaluate the awareness and perceptions of teledentistry among dentists. The study targeted 396 dentists, including general practitioners and pediatric dentists currently employed by the MOH in Kuwait. Ethical approval for the study was obtained from the Standing Committee for Coordination of Health and Medical Research, Ministry of Health, Kuwait (IRB #1896/2021).

The questionnaire, adapted and modified from a study by Al-Khalifa et al.,^17^ underwent a pilot test to ensure validity and reliability. It was subsequently distributed online between May and September 2022 through a WhatsApp link with one reminder, facilitated by the heads of the School Oral Health Program (SOHP) and the Pediatric Dentistry Department. This method ensured the inclusion of all dentists meeting the study’s inclusion criteria.

### Inclusion criteria

We included in the study all dentists managing pediatric patients in specialized dental centers, outpatient clinics for pediatric patients and SOHP clinics in the MOH in Kuwait. Dentists who manage pediatric patients and whose work is in emergency departments, private, academic, or military hospitals, Kuwait Oil Company Hospital, or as dental trainees and doctors on long leave were excluded.

The finalized questionnaire consisted of 34 questions divided into two sections. The first section included 11 questions focusing on demographic data (age, gender, qualifications, work experience, and work location). The second section contained 23 questions assessing participants’ awareness and perceptions of teledentistry.

Data were compiled in Microsoft® Excel® (2019) and analyzed using the Statistical Package for the Social Sciences (SPSS, version 2021). Descriptive statistics, including means and frequency distributions, were used to summarize the data. For inferential analysis, the Kruskal−Wallis test and the two-sample Wilcoxon rank-sum (Mann–Whitney) test were employed to compare mean scores across demographic variables when data distribution was non-normal. Statistical significance was set at *p* < 0.05. The distribution of data was assessed for normality using normality plots and the Shapiro-Wilk test, revealing a non-normal distribution. Consequently, the Kruskal−Wallis equality-of-populations rank test and the two-sample Wilcoxon rank-sum (Mann–Whitney) test were utilized to evaluate differences in mean response scores. For Likert scale questions, total responses for each answer were calculated, and the mean for each question was determined. These means were summed within each domain to derive response ranges: (3–9) for the first domain, with responses scored as 1 for “very concerned,” 2 for “little concerned,” and 3 for “not concerned at all”; and (6–24), (7–28), and (7–28) for the second, third, and fourth domains, respectively, where responses were scored as 1 for “agree,” 2 for “neutral,” 3 for “disagree,” and 4 for “I do not know.”

## Results

A total of 396 dentists working within the MOH and the SOHP were invited to participate in the survey. Of these, 106 respondents completed the questionnaire, yielding a response rate of 26.7%. All responses were deemed eligible for data analysis.

### Demographic and professional characteristics of respondents

The respondents had a mean age of 41.7 years. Females accounted for 66% of the participants, while males comprised 34%. Pediatric dentists formed the majority of the sample (65%), with the remaining 35% being general dental practitioners. Regarding professional experience, 29.2% of respondents had 11–25 years of experience, 28.3% had 10 years or less, and 13.2% had over 25 years of experience. Most respondents (58.4%) were employed in specialized dental centers, followed by 38.6% in SOHP, and only 2.8% in polyclinics (Table 1).

**Table 1.** Description of Demographic and Professional Characteristics of Participants

Variables	*N*	%
Age groups		
20–29 years	3	2.83
30–39 years	47	44.34
40–49 years	32	30.19
≥50 years	24	22.64
Gender		
Male	36	33.96
Female	70	66.04
Practice pediatric dentistry as		
Pediatric dentist	69	65.09
General dental practitioner	37	34.91
Work experience (in years)		
0–10 years	30	28.30
11–15 years	31	29.25
16–25 years	31	29.25
>25 years	14	13.21
Workplace		
Specialized dental center	62	58.49
Dental polyclinic	3	2.83
School oral health program	41	38.68
Location		
Al Asimah	19	17.92
Hawalli	18	16.98
Al Ahmadi	20	18.87
Farwaniya	24	22.64
Mubarak Al-Kabeer	2	1.89
Al Jahra	23	21.70

**FIG. 1.**
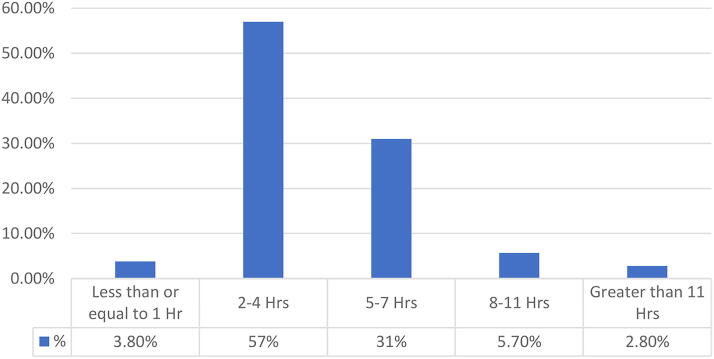
Number of Hours Spent on the Internet.

More than half of the respondents 57% reported spending 2–4 h daily on the internet (Fig. 1), while 75% spent 1 h or less using the internet specifically for dental practice purposes. A majority 77% believed that teledentistry is legally permitted in Kuwait, while 23% were uncertain or believed it to be illegal.

**FIG. 2.**
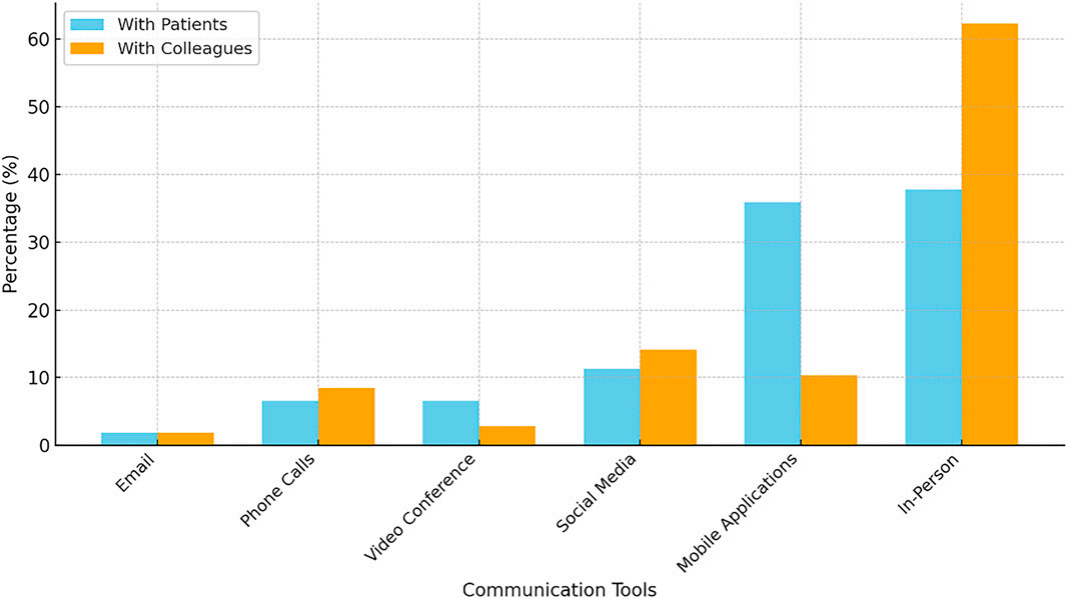
Preferred Communication Tools in Dental Practice.

Despite the availability of various digital communication tools, in-person interaction remained the preferred method of communication for both patients 37.7% and other dentists (colleagues) 62.2%. Among other methods, social media was used by 11.3%, video conferencing and phone calls by 6.6%, and emails by only 1.8%. Mobile applications emerged as the most preferred non-traditional communication tool with patients, though their overall adoption was limited. Advanced communication methods, such as social media and mobile applications, were not widely utilized for professional communication between dentists. This suggests a general preference for traditional methods over digital alternatives despite the growing accessibility of internet-based tools (Fig. 2).

### Dentists concerns relating to data security and patient consent

The data reveal significant concerns among dentists regarding the reliability of teledental equipment, patient consent, and data security in the context of teledentistry. Specifically, 44.34% of respondents expressed strong concerns about the reliability of teledental equipment, while 41.51% were highly concerned about obtaining patient consent for teleconsultation. Additionally, nearly half of the respondents 48.11% voiced substantial concerns about the online security of patient data. However, only a smaller proportion of respondents showed minimal concern (Table 2.) (Table 3).

**Table 2.** Concerns Regarding Data Security and Patient Consent

Concerns with regard to data security and patient consent	Very concerned *N* (%)	Little concerned *N* (%)	Not concerned at all *N* (%)
Reliability of teledental equipment	47 (44.34)	50 (47.17)	9 (8.49)
Confidentiality when data are sent online	51 (48.11)	46 (43.40)	9 (8.49)
Patient consent for teleconsultation	44 (41.51)	48 (45.28)	14 (13.21)

**Table 3.** Concerns Regarding Capabilities and Usefulness of Teledentistry

Topic	Very concerned/agree (%)	Neutral (%)	Little concerned/disagree (%)	Not concerned at all/I do not know (%)
**Capability to improve dental practice**				
Accurate diagnosis in clinical settings	15.09	54.72	19.81	10.38
Shortening waiting lists	70.75	16.98	7.55	4.72
Enhancing evidence-based dentistry	53.77	34.91	2.83	8.49
Improving peer interaction	59.43	29.25	3.77	7.55
Providing a safe atmosphere (e.g., COVID-19)	71.70	19.81	5.66	2.83
Making patient referrals more efficient	75.47	18.87	1.89	3.77
**Usefulness of teledentistry for dental practice**				
Enhancing clinical training and continuing education	75.47	17.92	2.83	3.77
Reducing costs for dental practices	54.72	30.19	10.38	4.72
Increasing appointment time spent with patients	38.68	27.36	18.87	15.09
Saving time compared with referral letters	79.25	13.21	2.83	4.72
Too expensive to set up	21.70	35.85	22.64	19.81
Providing adequate diagnostic information	30.19	35.85	20.75	13.21
Legally applied in Kuwait	23.58	8.49	11.32	56.60
**Usefulness of teledentistry for patients**				
Saving money for patients	54.72	32.08	2.83	10.38
Improving communication with patients	64.15	20.75	10.38	4.72
Helpful for patient education	89.62	9.43	0.00	0.94
Helpful for patients’ conditions	73.58	16.04	6.60	3.77
Convenient and well-received by patients	45.28	31.13	6.60	16.98
Useful for patients in remote areas	82.08	12.26	0.94	4.72
Beneficial for bedridden and compromised patients	78.30	14.15	3.77	3.77

### Perceptions of the capability of teledentistry to improve practice

A majority of respondents acknowledged the potential benefits of teledentistry, particularly in reducing patient waiting times 70.7%, integrating evidence-based dentistry 53.7%, improving peer interaction 59.4%, creating a safer environment 71.7%, and streamlining patient referrals 75.4% (Table 3). However, 54.7% were neutral regarding the accuracy of diagnosis through teledentistry, and fewer than 20% were uncertain or disagreed with its potential to improve practice.

### Usefulness of teledentistry for dental practice

Most respondents 75.4% agreed that teledentistry would enhance clinical training, save time compared with referral letters 79.2%, and reduce costs for dental practices 54.7% (Table 3). However, 38.6% believed it would increase appointment time with patients, while 18.8% disagreed. Many respondents 35.8% were neutral or unaware of teledentistry’s adequacy in providing diagnostic information or its setup costs. Knowledge about its legal application and usefulness in Kuwait was also limited.

### Usefulness of teledentistry for patients

Respondents generally agreed that teledentistry benefits patients by saving money, improving communication, aiding patient education, and being useful for remote areas (Table 3). However, 9.4–31.1% were neutral on these benefits, and 0.94–16.98% lacked knowledge on its usefulness for patients.

### Applications of teledentistry in pediatric dentistry

The majority of respondents acknowledged the potential applications of teledentistry in pediatric dentistry, particularly in enhancing dental education 91.5%, facilitating consultations 73.5%, and streamlining appointment scheduling 78.3%. However, 71% of respondents indicated that they found it challenging to effectively utilize teledentistry for dental diagnosis in pediatric patients. Despite these challenges, almost all respondents supported the integration of teledentistry into pediatric dental practice, recognizing its potential to improve accessibility and efficiency in certain areas of care.

Among the evaluated demographic variables and their associations with the four domains of teledentistry (concerns with data security, capability to improve dental practice, usefulness for dental practice, and usefulness for patients), only the practice in pediatric dentistry demonstrated a statistically significant difference. General dental practitioners had a higher mean score (10.73 ± 3.47) for the perceived capability of teledentistry to improve dental practice compared with pediatric dentists (9.16 ± 3.25, *p* = 0.0097), indicating greater confidence in its potential impact. For other demographic variables, including age, gender, work experience, workplace, and job location, no significant differences were observed across the four domains (*p* > 0.05), although variations in mean scores were noted. These findings emphasize that specialty influences perceptions of teledentistry’s capabilities, underscoring the need for tailored educational programs and interventions to address specialty-specific barriers and enhance the broader acceptance and integration of teledentistry in dental practice (Table 4).

**Table 4.** Statistical Analysis Between Dentists’ Demographics with the Four Domains of Teledentistry

Variable	Concerns with regard to data security	Capability of teledentistry to improve dental practice	Usefulness of teledentistry for dental practice	Usefulness of teledentistry for patients
	Mean (SD)	Mean (SD)	Mean (SD)	Mean (SD)
	4.96 (1.58)	9.71 (3.40)	14.02 (3.93)	10.34 (3.61)
Age (years)				
20–29 years	5.33 (2.31)	14.33 (4.16)	13.33 (5.13)	12.67 (4.04)
30–39 years	5.02 (1.47)	9.36 (2.35)	14.36 (3.64)	10.11 (3.23)
40–49 years	4.63 (1.50)	9.66 (2.96)	13.91 (3.46)	10.41 (3.32)
≥50 years	5.25 (1.82)	9.88 (5.02)	13.58 (5.00)	10.42 (4.66)
*p*-value^a^	0.5140	0.1235	0.8212	0.5403
Gender				
Male	5.17 (1.58)	10.17 (3.97)	13.53 (4.16)	9.58 (2.88)
Female	4.86 (1.58)	9.47 (3.07)	14.27 (3.81)	10.73 (3.90)
*p*-value^b^	0.2969	0.5521	0.5119	0.1255
Practice pediatric dentistry as				
Pediatric dentist	5.04 (1.59)	9.16 (3.25)	13.71 (3.70)	10.35 (3.95)
General dental practitioner	4.81 (1.58)	10.73 (3.47)	14.59 (4.32)	10.32 (2.94)
*p*-value^b^	0.5114	**0.0097**	0.1810	0.5646
Work experience (in years)				
0–10 years	4.80 (1.54)	9.77 (3.24)	14.70 (4.07)	10.70 (3.64)
11–15 years	5.16 (1.71)	9.32 (1.87)	14.16 (3.16)	10.03 (2.86)
16–25 years	4.61 (1.33)	9.65 (3.05)	14.13 (3.39)	10.19 (3.08)
>25 years	5.64 (1.74)	10.57 (6.25)	12.00 (5.71)	10.57 (5.88)
*p*-value^a^	0.2299	0.8697	0.0714	0.7973
Workplace				
Specialized dental center	5.08 (1.60)	9.08 (2.78)	13.95 (3.66)	10.05 (3.69)
Dental polyclinic	5.67 (2.52)	11.33 (6.81)	17.33 (1.53)	12.67 (3.79)
School oral health program	4.73 (1.48)	10.54 (3.83)	13.88 (4.37)	10.61 (3.49)
*p*-value^a^	0.5215	0.1318	0.1512	0.2548
Location of the main job				
Al Asimah	4.84 (1.42)	10.21 (2.72)	14.37 (4.25)	11.58 (3.67)
Hawalli	5.06 (1.89)	9.83 (4.20)	14.72 (4.11)	10.61 (2.89)
Al Ahmadi	4.90 (1.55)	10.00 (3.08)	14.10 (3.19)	10.70 (4.11)
Farwaniya	5.38 (1.47)	8.83 (2.16)	12.58 (3.55)	8.75 (2.20)
Mubarak Al-Kabeer	6.50 (3.54)	15.50 (12.02)	20.50 (10.61)	17.50 (14.85)
Al Jahra	4.48 (1.38)	9.35 (3.38)	14.04 (3.50)	9.83 (2.46)
*p*-value^a^	0.4149	0.5369	0.2715	0.0788

Bold font indicates significant *p*-value at 0.05 level of significance.

^a^ Kruskal–Wallis equality-of-populations rank test.

^b^ Two-sample Wilcoxon rank-sum (Mann–Whitney) test.

## Discussion

This study provides valuable insights into the awareness, perceptions, and potential barriers related to the adoption of teledentistry among pediatric dentists in Kuwait. To the best of our knowledge, this study is among the first to explore awareness and perceptions of teledentistry among dentists practicing pediatric dentistry in Kuwait. The response rate of 26.8%, representing nearly one-fourth of pediatric dentists in Kuwait, is comparable to similar studies in the region^17^ and reflects reasonable participation given the challenges associated with online surveys, such as potential response bias and variability in engagement.

The results of this study indicated a high level of positivity and agreement among respondents regarding the benefits of teledentistry in dental practice. Participants recognized the utility of teledentistry and its potential advantages for both patients and practitioners. These findings align with similar studies conducted in Australia and the Kingdom of Saudi Arabia, where dentists expressed strong support for the implementation of teledentistry, emphasizing its benefits for patients.^16–18^ However, these results contrast with previous research suggesting that postgraduates and interns exhibit lower awareness of teledentistry compared with first- and second-year dental students. This discrepancy may be attributed to the fact that teledentistry, being a relatively new concept, relies heavily on digital media that is more commonly utilized by younger generations. Furthermore, the limited awareness observed in older generations may stem from their lower exposure to evolving technological advancements.^14^

It was noteworthy that more than 50% of the respondents were unaware of the legal implications of teledentistry in Kuwait. While Decree #70/2020 was enacted to regulate telehealth services under the MOH, there has been insufficient clarity regarding its application in dental practice. This highlights the need for enhanced awareness among health care professionals, particularly dentists, through organized educational initiatives such as lectures and workshops aimed at providing a comprehensive understanding of the law and its implications for dental practice. In agreement with previous studies,^17,18^ the respondents showed high concerns about data security and patient consent, where 48% of them were concerned specifically about the confidentiality of the patient’s data to be sent online. Previous literature highlights similar concerns, including privacy, security, and financial issues related to electronic health care practices.^1^ However, advancements in technology have led to secure systems that protect patient data, addressing these concerns. Additionally, informed consent in teledentistry should include all elements of a traditional consent form, along with explanations of potential challenges, ensuring dentists can confidently manage patient confidentiality. The majority of participants 75% expressed strong agreement on the potential of teledentistry to enhance dental practice. Many published reports have similarly highlighted improvements in areas such as communication between dentists and colleagues, reduced waiting times, better delivery of evidence-based care, and more efficient patient referrals.^16,17^

Most dentists in our study recognized the benefits of teledentistry for patients, with 64.1% agreeing it improves communication, 89% finding it helpful for patient education, and 82% seeing its value for patients in remote areas and those who are bedridden or medically compromised. Literature highlights the advantages of teledentistry, especially for patients in remote areas facing access issues.^18^ While this is not a significant concern in Kuwait, where most dental facilities are within 10–40 min by car, the emergence of new remote residential areas makes teledentistry a valuable consideration for these populations.

### Applications of teledentistry

There were a high number of studies in the literature evaluating the application of Telehealth services in dentistry. Similar to previous studies, there was a general agreement among the participants about the application of teledentistry in consultation and in patient’s education and advice, which is considered one of the advantages of teledentistry in facilitating dental practice.^8,14,19–21^ Many respondents do not prefer applying this service in diagnosing dental diseases, as this was also reflected in multiple published studies.^9–12^ However, these studies considered teledentistry as a reliable mode of assessment for the diagnosis of dental diseases. This might be related to the accuracy of obtaining proper diagnosis through teledentistry and its effect in providing proper treatment compared with a clinical setting. Also, the patient should be informed regarding the possible risks of improper diagnosis and/or treatment due to the limitations of the technology involved. According to a recently published systematic review, pediatric dentistry and oral medicine were the most common dental specialty utilizing teledentistry in virtual asynchronous screening and diagnosis.^22^ Demonstrating good to very good accuracy in screening and diagnostic measures. This systematic review might increase the awareness among pediatric dentists to establish such services in their practice.^22^

### Study limitations

This study had several limitations. Unfortunately, in Kuwait, there is no contact database for health care workers, especially dentists that help researchers to retrieve any contact information to start their studies after obtaining ethical approval. In fact, there was no direct contact with the respondents, only through their team leaders. It was difficult to ensure that all potential participants received the online questionnaire link. In addition, the time the participants received the questionnaire link was highly dependent on the team leader, and that would have potentially affected the response rate. For example, in cases where the dentists received the invitation while they were busy at work, they will postpone the participation when they are free and hence, they might forget to respond to it. In addition, the timing of conducting this study (May–September 2022) was in the middle of summer holiday, where many dentists take their annual leaves around the school summer holidays, thus affecting the response rate. The online questionnaire has a lot of limitations mentioned in the literature, and it is highly dependent on the willingness of respondents to participate. On the contrary, if a hard copy of the questionnaire was distributed to the participants in person, they may feel impelled to participate. Notifications, messages, and phone calls received while filling out the questionnaire might interrupt the participants while they are responding to the questionnaire. The time of sending the reminder was delayed to ensure that more dentists were available after the summer holiday; however, if the reminder was sent closer to the initial invitation to participate this could have a positive impact in improving the response rate.

## Conclusion

This study highlights the growing importance of teledentistry in enhancing dental practice, particularly in response to the increased adoption of digital health solutions following the COVID-19 pandemic. While dentists in pediatric dentistry in Kuwait expressed a positive outlook on the potential of teledentistry to improve practice and patient care, the findings underscore the need for targeted educational initiatives to address specialty-specific barriers and promote broader integration of this technology across all dental fields. The study also emphasizes the necessity of raising awareness among dental professionals about the potential benefits and challenges of teledentistry, as well as the importance of developing clear regulatory frameworks to guide its implementation in dental practice. Future research should explore the perspectives of patients and other dental specialists to further assess the effectiveness of teledentistry and inform policy decisions. Ultimately, well-structured awareness campaigns and policy reforms will be crucial in ensuring the successful integration of teledentistry in dental practice, benefiting both health care providers and patients.

## Abbreviations Used

MOH: Ministry of Health
SD: Standard Deviation
SOHP: School Oral Health Program

## References

[1] Jampani ND, Nutalapati R, Dontula BS, et al. Applications of teledentistry: A literature review and update. J Int Soc Prev Community Dent 2011;1(2):37–44; doi: 10.4103/2231-0762.97695 24478952 PMC3894070

[2] Khan SA, Omar H. Teledentistry in practice: Literature review. Telemed J E Health 2013;19(7):565–567; doi: 10.1089/tmj.2012.0200 23672799

[3] Association AD. ADA Guide to Understanding and Documenting Teledentistry Events. Am Dent Assoc 2017;1–10.

[4] Golder DT, Brennan KA. Practicing dentistry in the age of telemedicine. J Am Dent Assoc 2000;131(6):734–744. 10860325 10.14219/jada.archive.2000.0272

[5] Kirshner M. The role of information technology and informatics research in the dentist-patient relationship. Adv Dent Res 2003;17(1):77–81. 15126213 10.1177/154407370301700118

[6] Mandall NA, Qureshi U, Harvey L. Teledentistry for screening new patient orthodontic referrals. Part 2: GDP perception of the referral system. Br Dent J 2005;199(11):727–729; discussion 723; doi: 10.1038/sj.bdj.4812969 16341186

[7] Bradley SM, Williams S, D’Cruz J, et al. Profiling the interest of general dental practitioners in West Yorkshire in using teledentistry to obtain advice from orthodontic consultants. Prim Dent Care 2007;14(3):117–122; doi: 10.1308/135576107781327061 17650390

[8] Stephens CD, Cook J. Attitudes of UK consultants to teledentistry as a means of providing orthodontic advice to dental practitioners and their patients. J Orthod 2002;29(2):137–142; doi: 10.1093/ortho/29.2.137 12114464

[9] Kopycka-Kedzierawski DT, Bell CH, Billings RJ. Prevalence of dental caries in Early Head Start children as diagnosed using teledentistry. Pediatr Dent 2008;30(4):329–333. 18767513

[10] Kopycka-Kedzierawski DT, Billings RJ. Teledentistry in inner-city child-care centres. J Telemed Telecare 2006;12(4):176–181; doi: 10.1258/135763306777488744 16774697

[11] Kopycka-Kedzierawski DT, Billings RJ. Prevalence of dental caries and dental care utilisation in preschool urban children enrolled in a comparative-effectiveness study. Eur Arch Paediatr Dent 2011;12(3):133–138; doi: 10.1007/BF03262794 21640057 PMC3111947

[12] Kopycka-Kedzierawski DT, Billings RJ, McConnochie KM. Dental screening of preschool children using teledentistry: A feasibility study. Pediatr Dent 2007;29(3):209–213. 17688017

[13] AlShaya MS, Assery MK, Pani SC. Reliability of mobile phone teledentistry in dental diagnosis and treatment planning in mixed dentition. J Telemed Telecare 2020;26(1–2):45–52; doi: 10.1177/1357633X18793767 30134778

[14] Boringi M, Waghray S, Lavanya R, et al. Knowledge and awareness of teledentistry among dental professionals–A cross sectional study. J Clin Diagn Res 2015;9(8):ZC41–ZC44. 26436045 10.7860/JCDR/2015/13303.6320 PMC4576639

[15] Pradhan D, Verma P, Sharma L, et al. Knowledge, awareness, and attitude regarding teledentistry among postgraduate dental students of Kanpur city, India: A questionnaire study. J Educ Health Promot 2019;8(1):104. 31143821 10.4103/jehp.jehp_363_18 PMC6532355

[16] Estai M, Kruger E, Tennant M. Perceptions of Australian dental practitioners about using telemedicine in dental practice. Br Dent J 2016;220(1):25–29. 26768465 10.1038/sj.bdj.2016.25

[17] Al-Khalifa KS, AlSheikh R. Teledentistry awareness among dental professionals in Saudi Arabia. PLoS One 2020;15(10):e0240825. 33057381 10.1371/journal.pone.0240825 PMC7561132

[18] Estai M, Kanagasingam Y, Tennant M, et al. A systematic review of the research evidence for the benefits of teledentistry. J Telemed Telecare 2018;24(3):147–156; doi: 10.1177/1357633X16689433 28118778

[19] AlKlayb SA, Assery MK, AlQahtani A, et al. Comparison of the effectiveness of a mobile phone-based education program in educating mothers as oral health providers in two regions of Saudi Arabia. J Int Soc Prev Community Dent 2017;7(3):110–115. 28584780 10.4103/jispcd.JISPCD_95_17 PMC5452563

[20] Lienert N, Zitzmann NU, Filippi A, et al. Teledental consultations related to trauma in a Swiss telemedical center: A retrospective survey. Dent Traumatol 2010;26(3):223–227; doi: 10.1111/j.1600-9657.2010.00873.x 20406276

[21] Snow MD, Canale E, Quail G. Teledentistry permits distant, cost-effective specialist dental consultations for rural Australians. J Telemedicine & Telecare 2000;6.

[22] Gurgel-Juarez N, Torres-Pereira C, Haddad AE, et al. Accuracy and effectiveness of teledentistry: A systematic review of systematic reviews. Evid Based Dent 2022:1–8. 10.1038/s41432-022-0257-8 PMC9264296 35804195

